# A Unified Framework for Creating Domain Dependent Polarity Lexicons from User Generated Reviews

**DOI:** 10.1371/journal.pone.0140204

**Published:** 2015-10-14

**Authors:** Muhammad Zubair Asghar, Aurangzeb Khan, Shakeel Ahmad, Imran Ali Khan, Fazal Masud Kundi

**Affiliations:** 1 Institute of Computing and Information Technology (ICIT), Gomal University, Dera Ismail Khan, Pakistan; 2 Institute of Engineering and Computer Science, University of Science and Technology, Bannu, Pakistan; 3 Faculty of Computing and Information Technology, King Abdul Aziz University (KAU), Rabigh, Saudi Arabia; 4 COMSATS Institute of Information Technology, Abbottabad, Pakistan; University of Illinois-Chicago, UNITED STATES

## Abstract

The exponential increase in the explosion of Web-based user generated reviews has resulted in the emergence of Opinion Mining (OM) applications for analyzing the users’ opinions toward products, services, and policies. The polarity lexicons often play a pivotal role in the OM, indicating the positivity and negativity of a term along with the numeric score. However, the commonly available domain independent lexicons are not an optimal choice for all of the domains within the OM applications. The aforementioned is due to the fact that the polarity of a term changes from one domain to other and such lexicons do not contain the correct polarity of a term for every domain. In this work, we focus on the problem of adapting a domain dependent polarity lexicon from set of labeled user reviews and domain independent lexicon to propose a unified learning framework based on the information theory concepts that can assign the terms with correct polarity (+ive, -ive) scores. The benchmarking on three datasets (car, hotel, and drug reviews) shows that our approach improves the performance of the polarity classification by achieving higher accuracy. Moreover, using the derived domain dependent lexicon changed the polarity of terms, and the experimental results show that our approach is more effective than the base line methods.

## Introduction

The continuous increase in the content of social media forums and online review sites has propelled the emergence of Opinion Mining (OM) applications. The users’ reviews available online about products, services, and policies assist consumers in their purchase decisions, and assist businesses to receive the clients’ opinions quickly [1]. The main focus of the studies in this area has been on issues, such as opinion detection [2], polarity classification at word [3], sentence and document level [4], [5], feature extraction [6], opinion summary generation [7], and polarity lexicon construction [8]. However, due to the growing interest in computing the exact polarity of terms within the OM applications, the polarity lexicon construction has become an active area of research [9].

Polarity lexicon construction deals with creation of large lists of words and phrases, where each word or phrase has positive, negative, or neutral polarity. The numeric score assigned to each of the word often represents the magnitude of the polarity [1], [3], [10], [11].There are three strategies for developing a polarity lexicon, namely: (i) manual, (ii) lexicon-based, and (iii) corpus-based [8]. The manual strategy is based on selecting and annotating the words manually by a group of experts. Such a strategy is costly in terms of time and the effort required for manual work. Moreover, there is a chance of omitting important words that could be included by other methods. The lexicon-based approach takes as an input an initial list of seed words and expands the list with the use of domain independent lexicon, such as SentiWordNet (SWN) [12]. The main disadvantage of such approach is that the final lexicon lacks the content and concepts needed for processing specialized information. The corpus-based approach can give sufficient coverage of such specialized content by learning the domain specific lexicon over a training corpus of labeled reviews in a specific domain. For example, the polarity of the word “heartbeat” is neutral (0.75) in the SWN. However, such a measure is inappropriate in the drug domain, e.g., in the sentence “*This drug is good enough as it normalizes my heartbeat*.” should have a positive polarity score. One possible solution for such problems is to modify the polarity of the words by using the corpus-based approach [8].

In this study, we explore the viability of domain dependent polarity lexicon that is created from the labeled reviews by adapting a domain independent polarity lexicon to a specific domain. We propose a method based on the information theory concepts, SWN, and improved feature weighting schemes. The proposed method is inspired by the previous studies performed on adapting domain independent lexicon to a domain dependent polarity lexicon [8], [13], [14], [15]. The previous studies [8], [13], [15], [16], [17] have used feature weighting and linear programming methods to adapt the domain independent lexicon to a domain dependent polarity lexicon to modify polarity of words over a limited set of labeled reviews. However, we use the SWN, information theory concepts, and feature weighting methods to adapt the domain independent polarity lexicon to a specific domain over a set of user generated reviews.

The main aim of the lexicon adaptation is to train the domain dependent polarities from the labeled reviews in a specified domain. To accomplish the above, we extract the polarity tendencies of the words by evaluating the mutual information with positive and negative reviews. When a particular word has more contribution in the negative reviews, compared to positive reviews, it is assumed to be of negative polarity, and vice versa. We also propose an improved weighting method for modifying a word’s polarity by using the MMI to quantify the polarity of a term, the frequency of a term (tf) within a document, and the inverse document frequency (idf).

The proposed methods enable us to modify the polarity of words on a larger scale. We demonstrate that such a large scale modification in the polarity of words has a significant role in improving the accuracy of the polarity classification. Our experiments show that the resulting lexicon is comparable to the existing lexicons in terms of accuracies obtained with the sentence level polarity classification. The above is an important issue in many OM applications, including opinion integration and summarization [18], [19], [20], [21]. We also perform accuracy-based comparison of the proposed weighting with other term polarity updation methods. The efficient data coverage of the proposed methods in specific domains is also presented to demonstrate the qualitative effectiveness.

A synopsis of the contribution is listed below.

1. The domain dependent lexicon is constructed by deriving it from domain independent lexicon over a set of labeled reviews.
2. Information theory concept: namely, Mutual Information (MI) is used to predict correct polarity class and score of domain dependent term.
3. Improved term weighting scheme is proposed and implemented for modifying polarity score of a word when there is a mismatch between SWN-based score and the newly computed score using MI.
4. The results obtained from the number of experiments conducted on term updating methods and polarity classification tasks using multiple datasets demonstrate the effectiveness of proposed approach.

The rest of the paper is organized as follows. Section 2 gives a review of related work. In section 3, we present methodology of proposed approach. Experimental setup is presented in section 4, which describes the datasets used, preprocessing, validation, evaluation metrics, and discussion on achieved results. The final section concludes the work with a discussion on the possible future work in this area.

## Related Work

The polarity lexicon plays an important role in most of the OM applications, such as opinion extraction [2], polarity classification [3], [4], feature extraction [6], and opinion summarization [7]. Even though the domain independent polarity lexicon have been shown to be effective for general purpose OM tasks [12], [16], [22], studies conducted by [8], [13], [14], [23], [24], [25],[26], [27] demonstrate that polarity of word changes with the change in domain. Therefore, more effective methods are required to develop a domain dependent polarity lexicon by adapting them from domain independent lexicon. We provide brief discussion of similar studies and identify the major differences compared to our contributions.

Many methods for developing the domain independent lexicons have been proposed in the recent past. Most of them rely on the existing lexical resources, such as Web documents, corpus of user generated reviews, and some existing lexicons. The polarity scores of words in such lexicons always take the value between 1 and -1. One of the popular OM polarity lexicons is the SWN [12]. The SWN is based on the WordNet database [10]. Each entry (synsets) within the SWN is given positive, negative, and objective scores within the range of 0.0 and 1.0, with the overall sum of 1.0. The Synsets relationship and the Gloss descriptions are used to evaluate the polarity of entries. In contrast to the work performed by [12], [22], Velikovich et al. [16] developed a polarity lexicon from a massive collection of Web documents using the graph propagation algorithm. The approach for the lexicon construction does not include the linguistic resources, such as WordNet and POS Tagger, rather the index terms are used to evaluate the polarity of terms with respect to the size and quality. The lexicon provides a sufficient coverage of both of the positive and negative phrases, covering spelling omissions, and vulgarity in the negative sentences expressed in social media posts. Another commonly used opinion lexicon is the OpinionFinder, compiled manually from various resources and learnt over a corpus [22]. The OpinionFinder is comprised of approximately 7,000 entries. The entries in the lexicon are labeled as strong and weak subjective, and each entry is also labeled as positive, negative, or neutral. A sample entry from the OpinionFinder takes the form of type = strongsubj word1 = avid, pos1 = adj, mpqapolarity = strongpos, which shows that the word “avid” when taken as an adjective is strongly subjective and the polarity is strongly positive. We are using the SWN-based scores from [12] in our approach for the polarity classification and later on, adapting the classification to specific domains.

The above mentioned approaches for the polarity lexicons are used for domain independent OM applications. However, there is no domain independent lexicon that can assign the correct polarity score to a word for every domain. This is due to the fact that the polarity score of a word is generally domain dependent [8] and changes with the change in the domain. It gives rise to development of domain adapted polarity lexicons with added functionality of words’ polarity modification.

The lexicon adaptation for different domains has improved the performance of various OM applications, such as opinion orientation [4], [9] and sentence level opinion classification [13]. To develop such lexicon, some of the studies have adapted the domain independent lexicon to domain specific by modifying the polarity of words using various approaches, such as tf x senti [8], tf x MI [17] and Delta Scoring [28]. Wilson et al. [29] emphasized the importance of context level polarity as compared to the prior polarity of a word. They defined numerous contextual features for extracting the contextual polarities. Goeuriot et al. [30] developed the polarity lexicon that deals with the health related OM. The resulting lexicon is obtained by merging the SWN and the Subjectivity Lexicon (SL) that is based on the patient feedbacks to constitute training and testing corpus. The polarity of domain specific words is changed by computing the corresponding information gain. A corpus oriented bootstrapping technique is proposed in [25] for creating a domain specific lexicon. The seed word list is dynamically updated by adding the best hypothesis. The authors applied the algorithm on the MUC-4 terrorism domain using eleven semantic classes. Based on the multiple linguistic resources, Souza et al. [31] proposed a domain dependent polarity lexicon for the Portuguese language. In the approach, three different polarity lexicons [1], [32] are adopted to construct a large and single opinion lexicon.

The main motivation of this work is the integer linear programming approach suggested by [13], which modifies the polarity of the words based on the word and expression level constraints. For example, if a word has a positive polarity in a domain independent lexicon but appears frequently in negative documents, then the polarity is modified to negative. In a recent work [8], the authors address the issues of adapting domain independent polarity lexicon to a domain dependent, and proposed a simple but an effective variant of the delta tf-idf weighting scheme.

**Fig 1:**
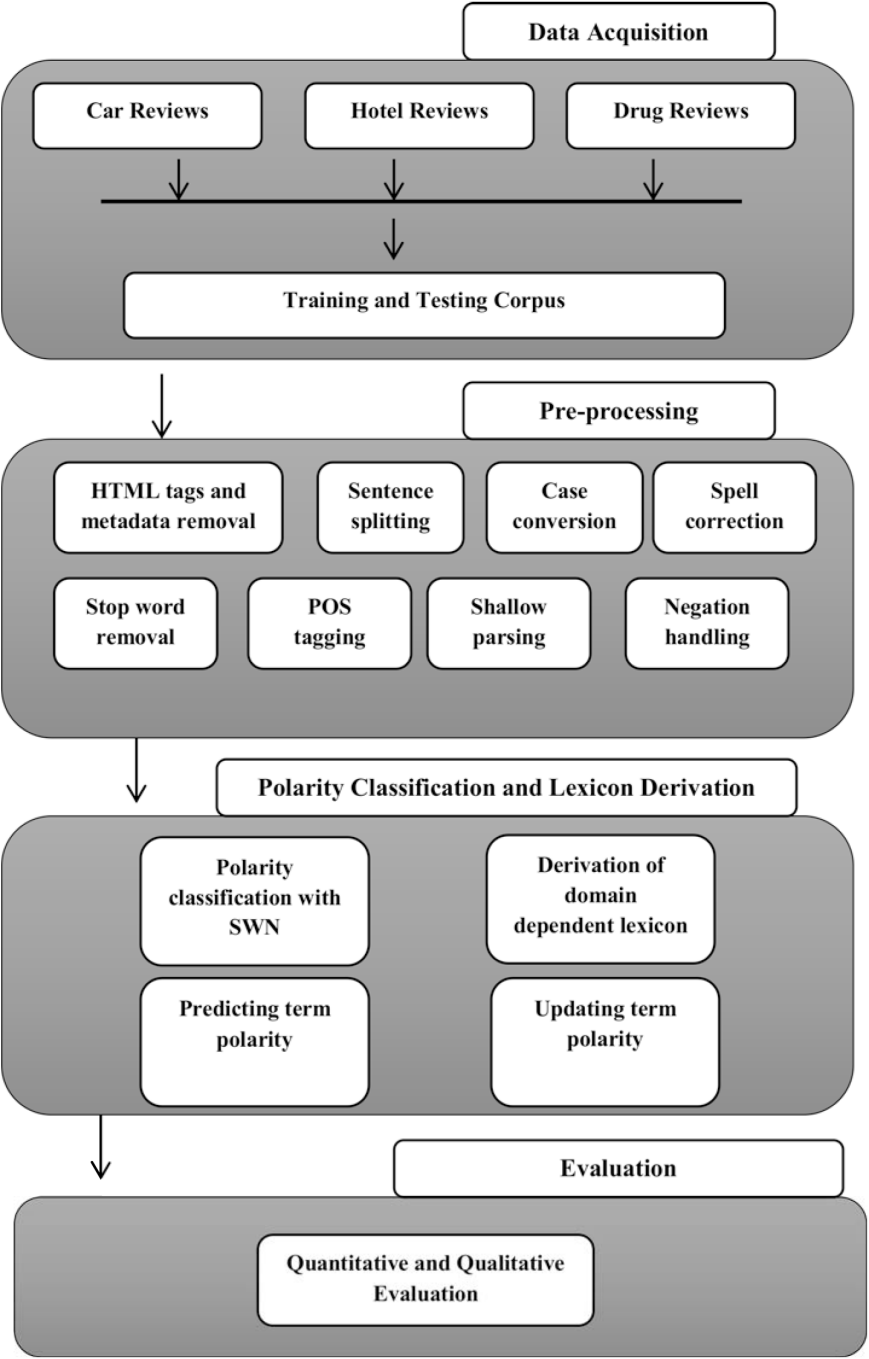
Detailed architecture of proposed framework.

More importantly, although the existing recent studies have improved the performance of OM applications, none provide a unified framework for combining domain independent, domain dependent polarity lexicons. Moreover, none address the need for mutual information based polarity modification scheme on lager datasets in different domains to improve the polarity classification at the term and sentence level, which is what we address in this work. The proposed framework is presented in Fig 1.

## Methods

In this section, we propose a method for adapting a domain independent polarity lexicon to a domain dependent lexicon. The method consists of three main steps: 1) first, we evaluate the polarity of terms using a domain independent polarity lexicon; 2) second, the polarity classification is performed by adapting a domain independent polarity lexicon to a specific domain by modifying the existing Mutual Information (MI) definition and 3) finally, the new polarity score is computed whenever there is a mismatch between the assignment from the domain independent lexicon and the "derived domain dependent lexicon" based on the authors' classification.

### Polarity Classification with Domain Independent Polarity Lexicon

To evaluate polarity of terms using a domain independent polarity lexicon, we choose to use SWN [12], because of its sufficient word coverage and frequent updates. The SWN is a domain independent lexical resource available publically with more than 60,000 terms (synsets) retrieved automatically from WordNet [10].

As a first step, we evaluate each entry of SWN. Let each entry in SWN is represented as a set of *V* terms:

$$V_{i}=<POS,swn.id,\;pol_{+},\;pol_{-,}\;pol_{o,}\;S,G>$$ (1)

where *POS* represents part-of-speech of the entry, *swn*.*id* is the SentiWordNet key, *pol*_*+*_, *pol*_*-*_,and *pol*_*o*_ are the positive, negative, and objective scores of *v*_*i*_ such that *pol*_*+*_ *+ pol*_–_ *+ pol*_*o*_ = 1, *S[V*_*i*_ *]* = *{s*_*o*,_ *s*_*1*_, *s*_*3*_, *…*., *s*_*n*_ *}* are the synsets of *V*_*i*_, and *G* is the gloss definition of *V*_*i*_.

Each term is associated with three polarity scores: positive, negative and neutral, represented as: <pol-, pol =, pol+>. These scores represent positivity, negativity and objectivity of each term respectively. The value of each score ranges in the interval from 0.0 to 1.0, and their overall sum equals 1.0 for each term. An example entry from SWN is represented as: <n, 7478318, 0, 0.75, 0.25, Syncope\#1 swoon\#1, faint\#1, deliquim\#1, a spontaneous loss of consciousness caused by insufficient blood to the brain>. This entry represents noun with the polarity scores 0.0, 0.75, and 0.25 (positive, negative and objective). It contains the terms Syncope, swoon, faint and deliquim, and WordNet gloss “*a spontaneous loss of consciousness caused by insufficient blood to the brain*”.

To compute polarity of a term, we choose its dominant polarity “*pol*_*swn*_(*V*_*i*_)” as:

$$pol_{swn}\left(V_{i}\right)=\{\begin{array}{cc} \;1 & \;if\;max\left(pol_{+},pol_{-},pol_{o}\right)=pol_{+}\\ \;-1 & \;if\;\text{max}\left(pol_{+},pol_{-},pol_{o}\right)=pol_{-}\\ \;0 & \;else \end{array}$$ (2)

The “*V*_*i*_” is positive if the +ive score is greater than both–ive and objective score. We get the–ive polarity by applying the same rule. The polarity is considered as objective if +ive and–ive polarities are equal or the objective polarity is greater than +ive and–ive. For example, the polarity window < *pol*_*+*,_ *pol*_–,_ *pol*_*o*_> for a term “Insomnia” is <0.0, 0.75, 0.25>; therefore *pol*_*swn*_ (“Insomnia”) = –0.75.

To evaluate the polarity of terms having multiple senses, we compute three mean values μ_+,_ μ_–,_ and μ_o_ for all the synsets of a term “*V*_*i*_” with respect to its part-of-speech (*POS*):

$$\mu_{+}\left(V_{i},POS\right)=\frac{1}{numSyn}\overset{n}{\underset{i=1}{\sum }}pol_{+}\left(i\right)$$ (3)

$$\mu_{-}\left(V_{i},POS\right)=\frac{1}{numSyn}\overset{n}{\underset{i=1}{\sum }}pol_{–}\left(i\right)$$ (4)

$$\mu_{o}\left(V_{i},POS\right)=\frac{1}{numSyn}\overset{n}{\underset{i=1}{\sum }}pol_{o}\left(i\right)$$ (5)

where +, -, and o represent the mean polarity score (positive, negative, objective) of synset *i* for term *V*_*i*_, *POS* denotes part of speech (noun, adjective, verb, and adverb), and numSyn is the total number of synsets of the term *V*_*i*_.

### Derivation of Domain Dependent Lexicon

In this section, we present our technique for the derivation of domain dependent lexicon. The proposed module updates the term’s polarity, if the occurrence of the term in training corpus represents one category (+ive or–ive) and SWN indicates the other category.

Let L be a set of training sentences defined as:

$$\text{L}={\text{l}}_{1}({\text{t}}_{1},....{\text{t}}_{\text{n}}).......{\text{l}}_{\text{n}\;(}({\text{t}}_{1},....{\text{t}}_{\text{n}})$$

Where *li* is the *i-th* training sentence and *t*_*j*_ is the *j-th* training term.

Let U be a set of testing sentences defined as:

$$\text{U}={\text{u}}_{1}({\text{t}}_{1},....{\text{t}}_{\text{n}}).......{\text{u}}_{\text{n}}{\;}_{(}({\text{t}}_{1},....{\text{t}}_{\text{n}})$$

Where *u*_*i*_ is the *i-th* testing sentence and *t*_*j*_ is the *j-th* testing term.

Our objective is to automatically predict the polarity class of all of the testing terms by computing Mutual Information (MI).

#### Mutual Information

The MI is a statistical technique used to measure the mutual dependence of two random variables by minimizing the uncertainty of one random variable on the basis of other random variable’s information. We compute the MI to measure the relationship between a term *t* and class label *c*, as:

$$MI\left(t,c\right)=\log\frac{p\left(t,c\right)}{p\left(t\right)p\left(c\right)}$$ (6)

Where *p*(*t*) and *p*(*c*) are the marginal probabilities of co-occurrence of term *t* and class label *c* respectively. The *p*(*t*, *c*) is the joint co-occurrence probability of term *t* and label *c*. An example 2 x 2 contingency table for this computation is shown below.

Table 1 presents co-occurrence values for terms and class labels. We observe that for N given labeled samples (N = A+B+C+D), A is the co-occurrence frequency of term *t* and class label *c*, B is the frequency of term *t* without class label *c*, and C is the number of sentences with class label *c* excluding the term *t*. Therefore the mutual information MI(*t*, *c*) of term *t* and class label *c* is approximated as:

$$MI\left(t,c\right)=\log\frac{A\;X\;N}{\left(A+B\right)X\;\left(A+C\right)}$$ (7)

The zero value of MI indicates that the term and class label are independent; whereas, higher the MI score, the greater the occurrence strength between *t* and *c*.

**Table 1:** Contingency table for term and class label.

	*t* (term occurs)	*t* (term does not occur)	
*c* (class label occurs)	A	C	A+C
$\bar{c}$ (class label does not occur)	B	D	B+D
	B+C	C+D	N = A+B+C+D

#### Predicting Term Polarity

To predict polarity inclination of term *t* with +ive or +ive class, we take two class labels, the +ive label *cp* and -ive label *cn*. The term *t* is +ive provided that the polarity score of *MI*(*t*, *c*_*p*_) is higher than the *MI*(*t*, *c*_*n*_). The polarity score *Pol*_*MI*_(*t*, *c*_*p*_) of term *t* on class label *c*_*p*_ is formulated from the linear combination of *MI*(*t*, *cp*) and *MI*(*t*, *cn*) as:

$$Pol_{MI}\left(t,c_{p}\right)=\beta MI\left(t,c_{p}\right)+\left(1-\beta \right)\left(-MI\left(t,c_{n}\right)\right)$$ (8)

Similarly, we compute polarity score *Pol*_*MI*_ (*t*, *c*_*n*_) as:

$$Pol_{MI}\left(t,c_{n}\right)=\beta MI\left(t,c_{n}\right)+\left(1-\beta \right)\left(-MI\left(t,c_{p}\right)\right)$$ (9)

Where 0 ≤ β ≤ 1 and it is the threshold reflecting the contributions of *MI*(*t*, *c*_*p*_) and *MI*(*t*, *c*_*n*_).

To evaluate the contribution of term *t* in global feature space, we integrate *Pol*_*MI*_(*t*,*c*_*p*_) and *Pol*_*MI*_(*t*,*c*_*n*_) as:

$$Pol_{MI}\left(t\right)=\{\begin{array}{l} Pol_{MI}\left(t,c_{p}\right)\;if\;Pol_{MI}\left(t,c_{p}\right)>Pol_{MI}\left(t,c_{n}\right)\\ Pol_{MI}\left(t,c_{n}\right)\;if\;Pol_{MI}\left(t,c_{n}\right)>Pol_{MI}\left(t,c_{p}\right)\\ \;0\;if\;Pol_{MI}\left(t,c_{p}\right)=Pol_{MI}\left(t,c_{n}\right) \end{array}$$ (10)

If *Pol*_*MI*_ *(t*, *cp) > Pol*_*MI*_ *(t*, *cn)* holds, then the polarity of the term *t* is positive, and the accumulative *Pol*_*MI*_ *(t)* polarity score is considered to be positive. Conversely, if the polarity of term *t* is negative, then the *Pol*_*MI*_ *(t)* score will be negative. For example, taking *Pol*_*MI*_ *(t*, *cp)* = 7 and *Pol*_*MI*_ *(t*, *cn)* = 0.4, the term *t* tends to be positive and *Pol*_*MI*_ *(t)* = 7. If the value of *Pol*_*MI*_ *(t*, *cp)* is less than that of the *Pol*_*MI*_ *(t*, *cn)* then the term t indicates a negative polarity. In the latter case, if the polarity scores of the two class labels are equal then the polarity of the term *t* cannot be identified. Therefore, the proposed technique can assign the correct polarity label to a term in a testing corpus.

We take another case: the term *t* occurs 3 times in one negative review without appearing in the rest of the negative reviews. In the meantime, the term *t* occurs once in each of the positive reviews. Therefore, the term *t* tends to be positive. In our method, the value of *Pol*_*MI*_ *(t*, *cp)* is larger than that of *Pol*_*MI*_ *(t*, *cn)*, which indicates that the term *t* is more inclined to positive class. Therefore, our technique can acquire the accurate polarity tendency of terms in the corpus.

Table 2 shows a sample list of unigram and bigram from our car and drug dataset along with SWN-based polarity and predicted polarity. We can see that most of the terms reflect accurate polarity inclinations. The bigram “not_helpful” indicates that a negation “not” precedes the term “helpful”, which will be discussed in section “Pre-Processing”. When someone is not satisfied with some drug, he can often express his sentiment as: “*I was using Medrol for the last one week for root canal issue*, *but it was not helpful for migraine”*. Therefore, we need to handle negations properly. There are certain outliers, such as, “Toyota” and “GLI” etc., which will be discussed further in section “Results and Evaluation”.

**Table 2:** Sample list of positive and negative unigram and bigram in drug and car domains.

Drug Domain			Car Domain		
Word	SWN Polarity	Predicted Polarity using Eq 10	Word	SWN Polarity	Predicted Polarity using Eq 10
bathe	Neutral	Positive	Drive	Neutral	Positive
recovery	Neutral	Positive	GLI	Not found	Positive
anti-Inflammatory	Neutral	Positive	ride	Neutral	Positive
child-proof	Neutral	Positive	XLI	Not found	Positive
safe dose	Not found	Positive	air bags	Not found	Positive
frozen shoulder	Not found	Negative	reading lights	Not found	Negative
growth	Neutral	Negative	repair	Neutral	Negative
Stomach	Neutral	Negative	price	Neutral	Negative
Move	Neutral	Negative	cold starting	Not found	Negative
day blindness	Not found	Negative	grinding noise	Not found	Negative
not_helpful	Not found	Negative	not_working	Not found	Negative

### Updating Term Polarity

When there is mismatch between the SWN-based average scores (Eq 3, Eq 4 and Eq 5) and the *Pol*_*MI*_ *(t)* score of a term (Eq 10), we consider updating its polarity. For example, the term “growth” has neutral polarity in SWN, while the corresponding *Pol*_*MI*_ *(t)* score is negative, indicating that it has more inclination with the negative class. Similarly, the term “bathe” has a neutral polarity in SWN; while it’s *Pol*_*MI*_ *(t)* score is positive, indicating that it has more tendencies in positive class.

For computing new polarity of a term where a mismatch is found, we combine the term frequency (*tf*), inverse document frequency (*idf*) and the polarity score *Pol*_*MI*_ *(t)*.Therefore, the updated polarity score *Pol*_*new*_ *(t*, *r)* for term *t* in review *r* is defined as:

$$Pol_{new}\left(t,r\right)=tf\left(t,c\right).idf\left(t\right)\;X\;Pol_{MI}\left(t\right)$$ (11)

where *tf (t*, *c)* is frequency of term *t* in class *c* and *idf(t)* is the proportion of reviews where the term *t* occurs, ignoring high frequency words in the review corpus (e.g. ‘the’, ‘not’, ‘is’, ‘be’).

As can be observed in Table 3, the proposed measure (Eq 11) has updated the polarity score of the term “move” in the drug dataset. The term “move” appeared in a sentence like “*He found he was unable to move*”, where it reflects to negative sentiment. The SWN-based dominant polarity score of the “*move*” was 0.975 and the updated polarity score is -3.5.

**Table 3:** Words and their polarity coverage.

No.	Word	SWN Score (Eqs 3, 4 and 5)	tf(t, c). idf(t) x Pol_MI_ (t) (Eq 11)	Example Sentence	Dataset
1	bathe	1 (neutral)	3.5 (positive)	He bathed the grazed knee with boiled water.	Drug
2	relax	0.625 (neutral)	3.0(positive)	alpha blocker can relax muscle, to treat urinary retention and hypertension	Drug
3	growth	1 (Neutral)	4.5(Negative)	The doctor found a cancerous growth on the left breast.	Drug
4	drive	1 (neutral)	2.6(Positive)	My new 2015 Camry is great! it’s fun to drive	Car
5	hospital	0.8125 (neutral)	4.42(Negative)	He is in hospital with an infectious tropical illness.	Drug
6	move	0.975 (Neutral)	3.5(Negative)	He found he was unable to move	Drug
7	brakes	0.782(Neutral)	2.2(Negative)	Sentra really not as reliable in brakes especially in wet conditions	Car
8	Toyota	1(Neutral)	2.6 (positive)	Toyota Camry is very dependable and comfortable car.	Car
9	cruise control	not found	3.0 (negative)	Jeep Grand Cherokee 2013 has a great drive but the cruise control is horrible. when you set the cruise on 60 it will vary from 55 to 65 up and down hill	Car
10	ride	1(Neutral)	1.5(positive)	Quiet and soft ride which made us go to the Mercedes	Car

#### MI-based Algorithm

Algorithm 1 provides the pseudo code of the steps of the proposed MI-based technique for performing the polarity computation task.

### Algorithm 1. MI-based sentiment polarity computation

• **Input:**

*L*: dataset; *SWN*: Lexicon

• **Output:** The updated polarity *Pol_new(t*_*i*_ *)*

1. **for each** term *t*_*i*_ occurring in L **do**

    # Compute SWN based average score using Eqs (3), (4), and (5)

2. Calculate *Avg_SWN_Score*(*t*_*i*_ *)*

    # Compute *pol (t*, *c*_*p*_ *)* and *pol (t*, *c*_*n*_ *)* using Eqs (8) and (9)

3. **for** each class label c_i_ in {+, -} **do**

        Calculate *pol (t*, *c*_*p*_ *)* and *pol (t*, *c*_*n*_ *)*

    # Compute *Pol*_*MI*_ *(t*) score using formula (10)

4. **If** *Pol*_*MI*_ *(t*, *cp) > Pol*_*MI*_ *(t*, *cn)*

5. *Pol*_*MI*_ *(t*_*i*_) ← *Pol*_*MI*_ *(t*, *cp)*

6. ***else if*** *Pol*_*MI*_ *(t*, *cn) > Pol*_*MI*_ *(t*, *cp)*

7. *Pol*_*MI*_ *(t*_*i*_)← *Pol*_*MI*_ *(t*, *cn)*

8. ***else if*** *Pol*_*MI*_ *(t*, *cp) = Pol*_*MI*_ *(t*, *cn)*

9. *Pol*_*MI*_ *(t*_*i*_)←0

    **#** In case of mismatch between polarity scores calculated at step#2 and step#3–9, update polarity of *t*_*i*_ using formula(11)

10. *Pol*_*new*_ *(t*_*i*_ *)*← Calculate(*tf(t*_*i*_, *c*_*i*_ ) * idf (t_*i*_ ) * Pol_*MI*_ *(t*_*i*_ *))*

11. ***return*** *Pol*_*new*_ *(t*_*i*_ *)*

## Experimental Setup

We implemented all of the algorithms presented in section “Updating Term Polarity” and section “Results and Evaluation” using python and Natural Language Toolkit (NLTK) [33]. The lemmatization features were also incorporated using the NLTK library to boost the performance of the proposed algorithms. To implement, test, and evaluate the effectiveness of proposed approach, multiple datasets [34] were used to conduct the experiments. This study did not involve any experimental research on humans or animals; hence an approval from an ethics committee was not applicable in this regard. The data collected from the online forums are publicly available data and no personally identifiable information of the forum users were collected or used for this study. The following sections provide details of the datasets, experiments and results.

### Datasets

To validate the practical usefulness of the proposed approach, we use three publically available datasets, namely: (i) car reviews (C), (ii) hotel reviews (H), and drug reviews (D). Car and Hotel reviews are available at: https://archive.ics.uci.edu/ml/datasets/OpinRank+Review+Dataset, whereas drug reviews are obtained from publically available dataset (available at http://ir.cs.georgetown.edu/data/adr/).AlchemyAPI (http://www.alchemyapi.com/api *)* is used to classify reviews into positive, negative and neutral polarity. Such information is stored in a separate text file to constitute the entire corpus. We divide the corpus into a training dataset and a testing dataset, and store them in separate text files.

The training dataset consists of 7,200 reviews with 47% positive, negative and 6% neutral reviews. The testing dataset includes the remaining of the 16,800 reviews, with 53.33% positive, 36.66% negative, and 10% neutral reviews. During the training session, we systematically include the training samples until the completion of the entire training dataset, and analyze the performance on the testing dataset.

### Pre-Processing

The data is cleansed by passing through the pre-processing module as:

1. Html parser [30] is used to extract clean contents into text files by removing the html tags and the unnecessary metadata information.
2. We identify the sentence boundaries by breaking the cleaned text into sentences. Case-conversion, spelling correction, and stop words removal is performed with the help of frequently used stop-word list [17].
3. To obtain the sentence structure, all of the corpus sentences are parsed by using the Stanford parser [35]. The parser assigns *POS* tags to each of the word in sentence. In addition to full parsing, we also perform shallow parsing [8] on each of the sentence to split the sentence into group of related phrases, such as noun phrase, verb phrase, and prepositional phrase.
4. As identified in [36], stemming is not applicable as it degrades the accuracy of classification.
5. As presented in the work performed by [17], we discard the negation terms from reviews by appending the tag “not_” to the terms following the negation terms in a review. For example, the review “the pain killer didn’t work effectively.” can be changed to “the pain killer not_work not_effectively”.

### Validation

For supervised machine learning algorithm, we need two distinct parts of dataset, namely: training and testing. The two separate parts of one dataset ensures the true evaluation of the methods’ performance. Therefore, during each run, more than one run of the experiments are usually required with distinct datasets. We ran the experiments by using a special case of the N-fold cross validation, called Leave-One-Out-Cross Validation (LOOCV) [37]. The process consists of splitting the dataset into *N* folds (5–10 folds), where *N -*1 folds are used for training and the remaining one is used for testing the system. In such a case, we used the 5-fold cross validation. For each run, 4 of the five folds are used for training, and one for testing. The performance of classifier is estimated by taking average over all 5 experiments.

There are two types of features used commonly in polarity classification, namely: unigram and bigram, which we use for comparing the proposed technique with other polarity updating approaches, including the Delta-Scoring, tf x MI, and tf x senti.

- Delta-Scoring: It computes a new polarity based on (Δtf) idf score of a term [28].
- tf x MI: It assigns a new polarity based on term frequency and mutual information [17].
- tf x senti: It assigns a polarity based on Term frequency and SentiWordNet [8].
- tf x idf x MI (our) It assigns updated polarity based on computation performed in Eq 11.

### Evaluation Metrics

We use precision, recall, F-score, and accuracy for measuring the performance of the proposed technique in our experiments because they are considered as benchmark metrics for evaluating performance of classifier.

The precision, recall, F—score, and accuracy are computed as:

$$Precision=\frac{TP}{TP+FP}$$ (12)

$$Recall=\frac{TP}{TP+FP}$$ (13)

$$F-measure=\frac{2\left(precision\right)\left(recall\right)}{precision+recall}$$ (14)

$$\text{Accuracy}=\frac{TP+TN}{\text{TP}+\text{FP}+\text{TN}+\text{FN}}$$ (15)

where, True Positive TP is the number of positive reviews correctly classified, False Positive FP is the number of negative reviews incorrectly classified as a positive, True Negative TN is the number of negative reviews correctly classified, and False Negative FN is the number of positive reviews incorrectly classified as a negative are shown in Table 4.

**Table 4:** Different combinations of actual and predicted parameters.

Data Class	Actual	Predicted
Positive	TP	FN
Negative	FP	TN

## Results and Evaluation

In this section, we elucidate the quantitative results with qualitative analysis obtained from the experiments to evaluate the effectiveness of the proposed approach by using various evaluation metrics.

The First experiment was conducted to evaluate the effect of sentiment score *pol*(*t*) computed in Eq 11. Recalling the results presented in Table 3, almost all of the top 10 polarity scores of positive and negative words and phrases in drug and Car domain reflect correct polarity orientation. There are some outliers which need to be analyzed. The term “Toyota” always depict neutral polarity without context. However, the “Toyota” is a leading automobile manufacturer having corporate credibility among its customers. There are 25 reviews about the Toyota products in our dataset, and 95% of them are positive. Therefore, the term “Toyota” has strong inclination with positive class.

Eight of the eleven reviews on “ride” are marked as positive, and the remaining three are labeled as negative. Therefore, the term “ride” has more tendency towards positive class. As in the previous case, it is enough to observe that the terms “relax” and “hospital” have strong inclinations towards positive and negative classes respectively. Because the former is included in 18 positive reviews and 2 negative reviews and the later occurs in 10 negative reviews and in 2 positive reviews.

Recalling the results in Table 2, the terms “GLI” and “XLI” are the two brands of an automobile company whose vehicles gained remarkable recognition in our dataset. The most of the reviews on drugs refereeing to the term “anti-Inflammatory” are positive (20 positive and 4 negative). The term “stomach” in SWN has neutral polarity, whereas it appears frequently in the negative reviews of our drugs dataset (3 positive and 20 negative), such as “stomach problem”. Therefore, the term “stomach” is considered in the negative class. Therefore, our technique captures correct sentiment inclinations of terms in given datasets.

**Fig 2:**
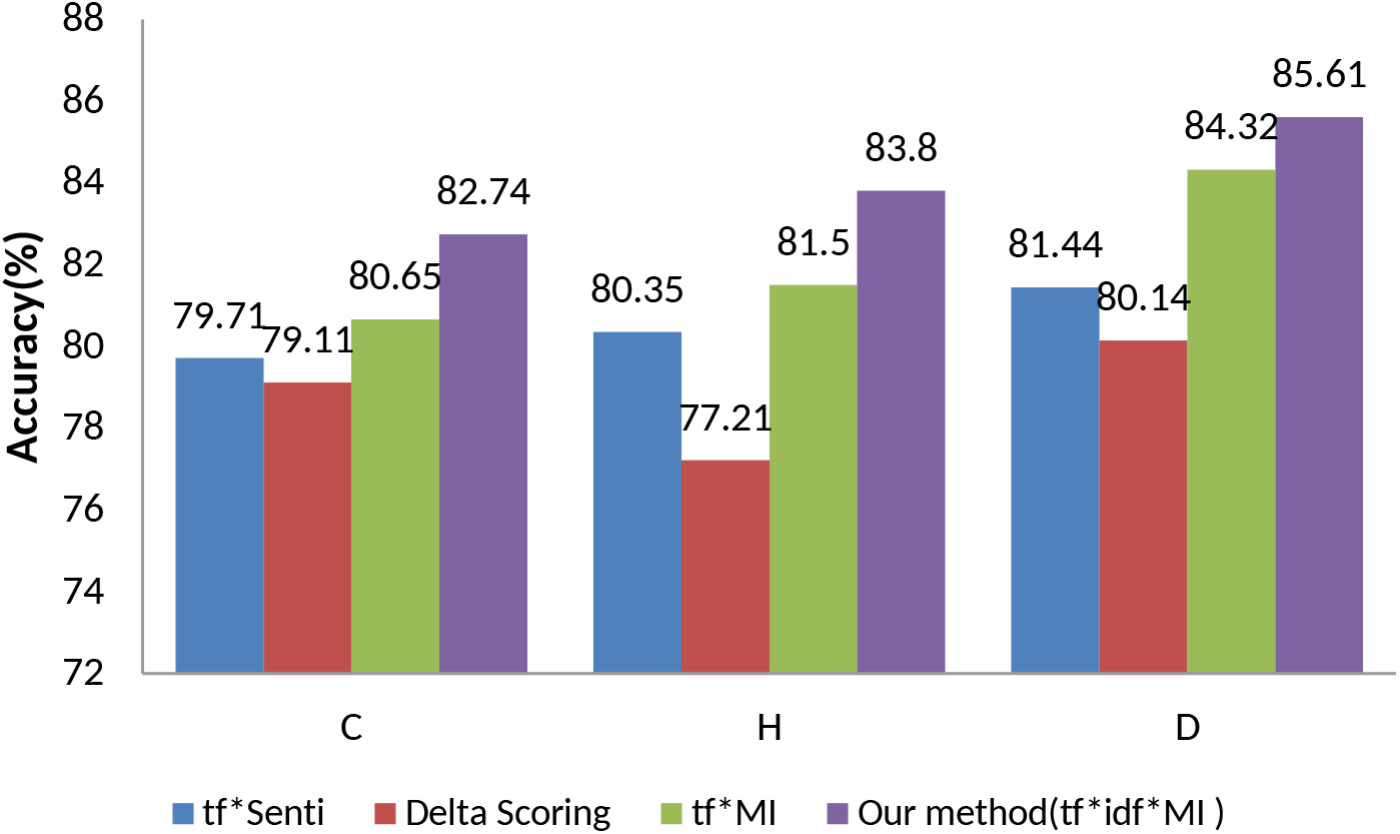
Unigram-based accuracy comparison of the proposed method with baseline methods.

**Fig 3:**
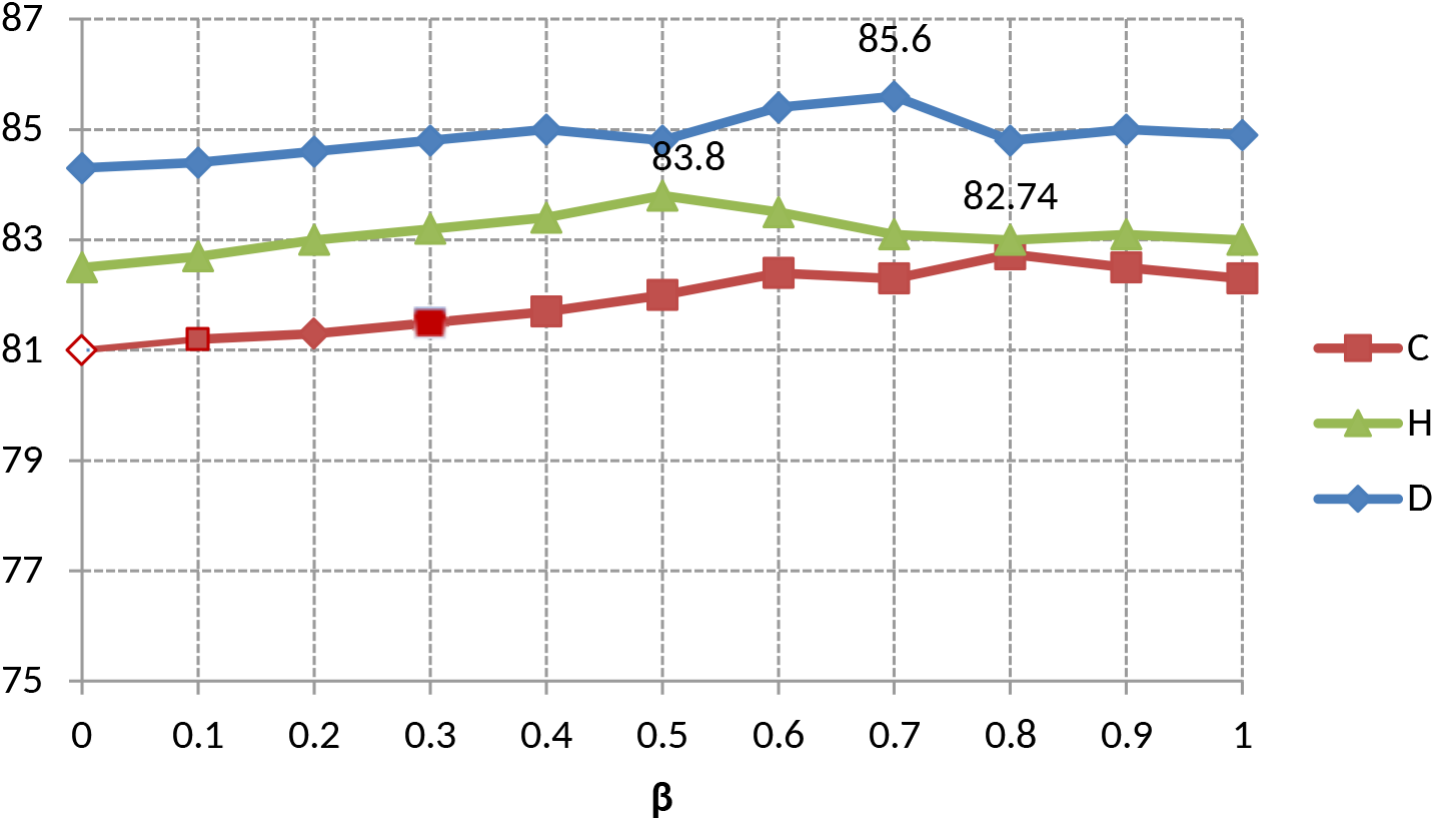
Unigram-based accuracies of the proposed method (tf x idf x MI) w.r.t varying value of β.

The second experiment was conducted to evaluate the effectiveness of tf x idf x MI based on the unigram. The Fig 2 shows the accuracy-based comparisons of the three datasets. The value of β on unigram for each dataset is shown in Fig 3. Our technique, tf x idf x MI, achieves the best performance for all datasets. The tf x MI and tf x idf x MI outperforms the other two polarity updating methods consistently. The accuracy of tf x idf x MI is about 2.66% higher than that of tf x MI in the three datasets. On the other hand, the delta scoring gives poor performance in all of the datasets due to multiple senses and meanings of a given word in a document. The tf x MI performs better than delta scoring on the average accuracy in the three datasets.

**Fig 4:**
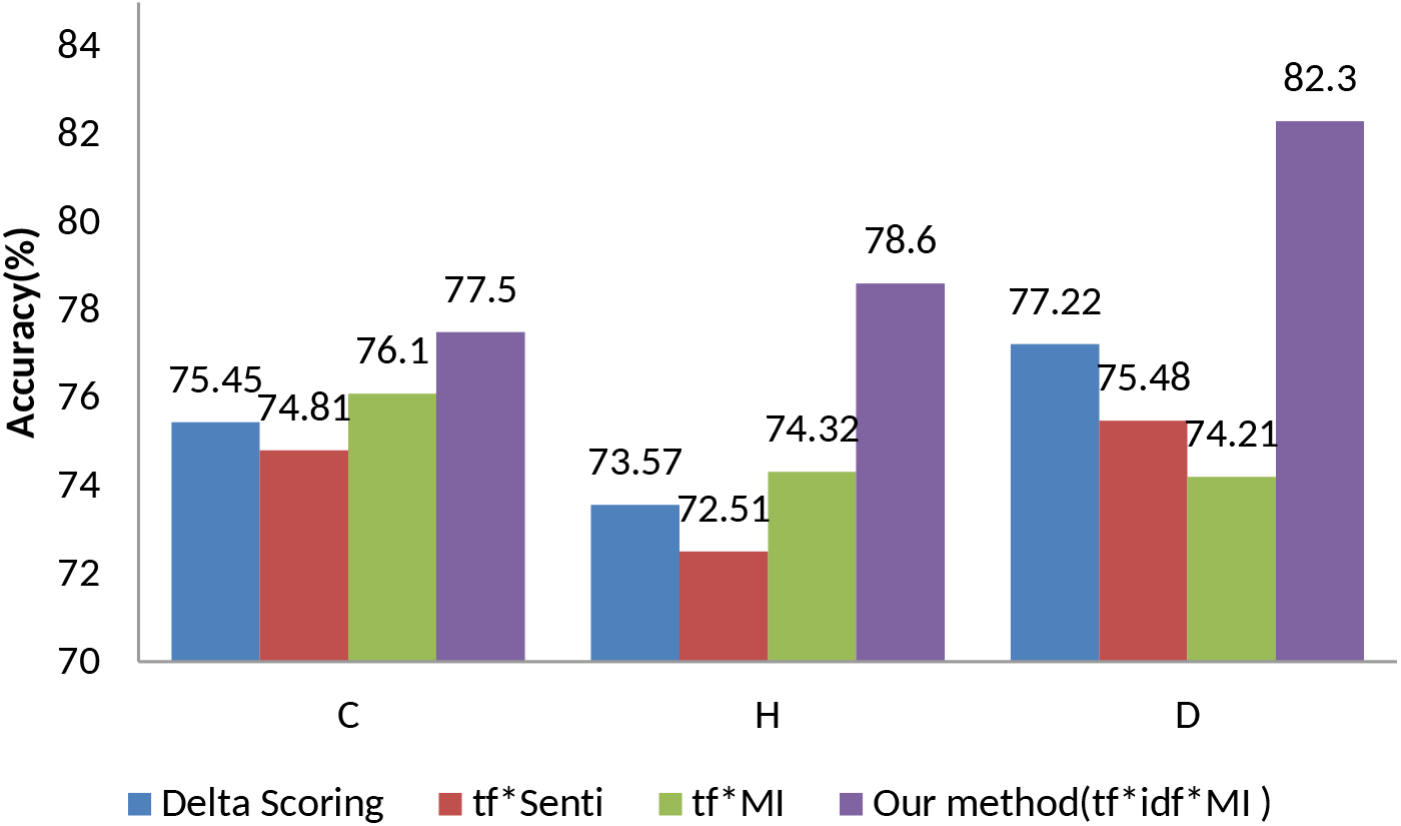
Bigram-based accuracy comparison of the proposed method with baseline methods.

**Fig 5:**
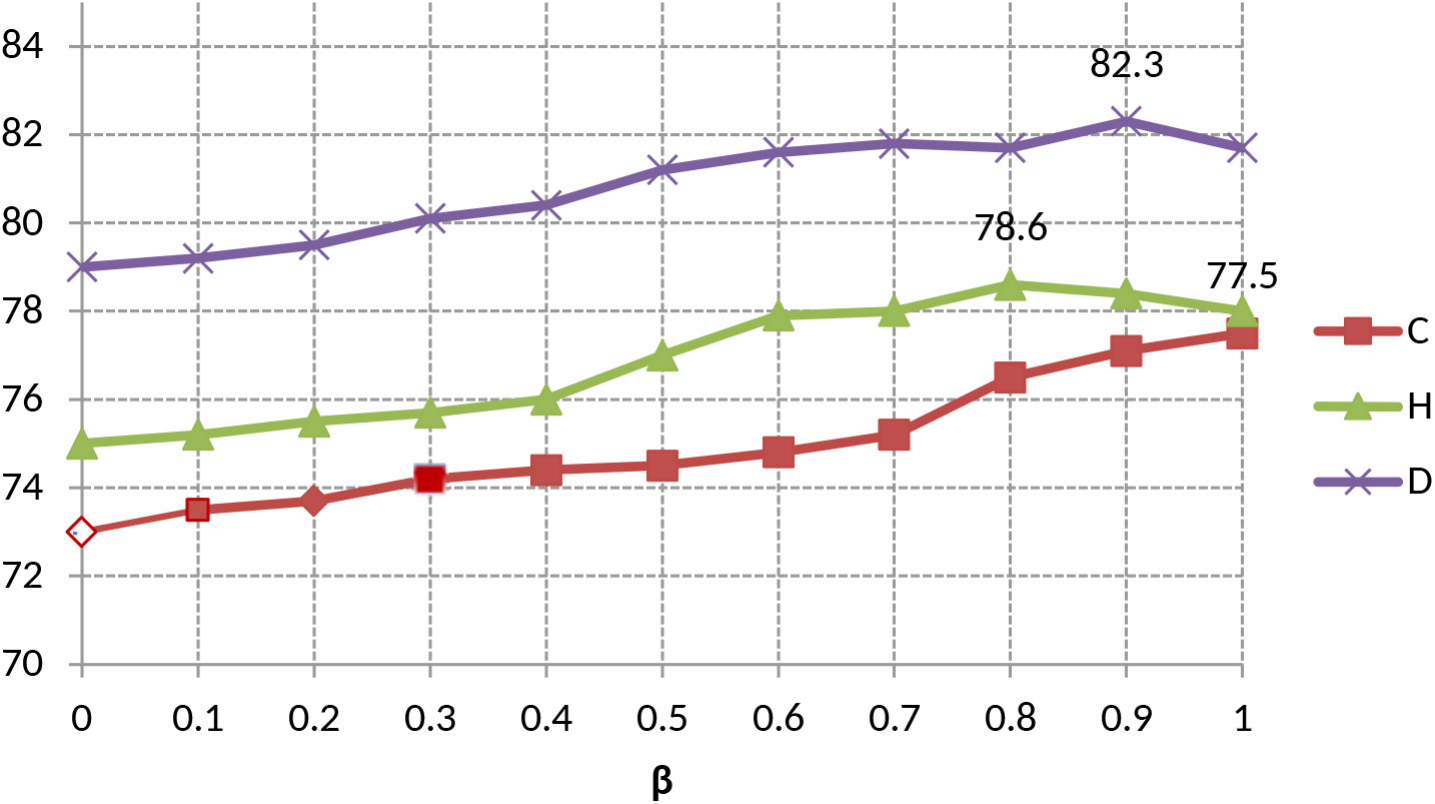
Bigram-based accuracies of the proposed method (tf x idf x MI) w.r.t varying value of β.

In Fig 4 we observe the comparisons on the polarity updating methods for bigram. The value of β on bigram for each dataset is shown in Fig 5. In this experiment, our technique, tf x idf x MI, achieves promising results in all datasets with improved accuracy. It is worth mentioning that the tf x MI shows poor performance than tf x senti and delta scoring for bigram in Drug reviews. Our technique achieves significant performance over the comparing methods in all datasets.

Moreover, we observe that classification accuracies on drug reviews based on unigram and bigram are higher than those in car and hotel reviews. The opinions expressed on drugs are often more pragmatic than those of other products. For example, a review of a drug is written as: “While taking this drug, I felt immense relief in cyanosis; I love its pleasant taste.” The terms “immense”, “relief”, “love”, and “pleasant” express highly positive sentiments. Similarly, the negative opinions expressed on a drug, such as “Loopy feeling, tired, very thirsty, when I take this drug”. The terms “Loopy”, “tired”, and “very thirsty” express intensive negative polarity opinions.

Finally, we observe that the effect of changing the value of β on unigram and bigram respectively. As described earlier, the polarity score of term *t* for class label *c* is the linear combination of MI with a positive class label *c*_*p*_ and a negative positive class label *c*_*n*_. Fig 3and Fig 5 show that tf x idf x MI achieves best results when the value of β ranges between {0.4 and 1}. For the unigram, the value of β should be within {0.4 to 0.8}, and for bigram, it ranges from 0.7 to 1.0. Overall, our technique achieves the higher accuracy results as compared to all of the other techniques.

The fourth experiment is conducted to measure the practical effectiveness of our derived polarity lexicon on the sentence polarity classification task. We used the vote-flip algorithm [30] for calculating the polarity of sentences by classifying them as being positive, negative, and neutral. Such a basic algorithm (Algorithm 2) provides a simple mechanism to evaluate our lexicon.

The algorithm keeps track of the matched positive and negative unigram and bigram from the lexicon and whichever has the maximum votes is deemed the winner. The algorithm flips the output if there are an odd number of negations.

As we are considering documents (reviews), we applied the algorithm at the document level to a corpus of 24,000 reviews, among which, 7,200 were training reviews and 16,800 were test reviews. A document/review is negative, if it contains most of the negative sentences. We applied the algorithm to the user reviews for labeling them as positive, negative and neutral.

### Algorithm 2. Vote Flip Algorithm

• **Input:**

            Lexicon L

            Sentence S

            Negation terms NEG

• **Output:**

Polarity = {POSITIVE, NEGATIVE, NEAUTRAL}

1. **for each** term t in S

2. set *pos*, *neg*, *ngt* = 0 // term opinion count

3. **If** pol_L_ > 0 **then** *pos++*

4. **else if** pol_L_<0 **then** *neg—*

5. **else** *if t Ɛ NEG* **then** *ngt++*

6. flip = (ngt mod 2 = = 1) // check if *ngt* is odd

7. **if** (*pos>neg and not flip)* **or** *(neg>pos and flip)* **then**

8. *return* POSITIVE

9. **else if** (*pos>neg and flip)* **or** *(neg>pos and not flip)* **then**

10. *return* NEGATIVE

11. **else**

12. *return* NEAUTRAL

**Fig 6:**
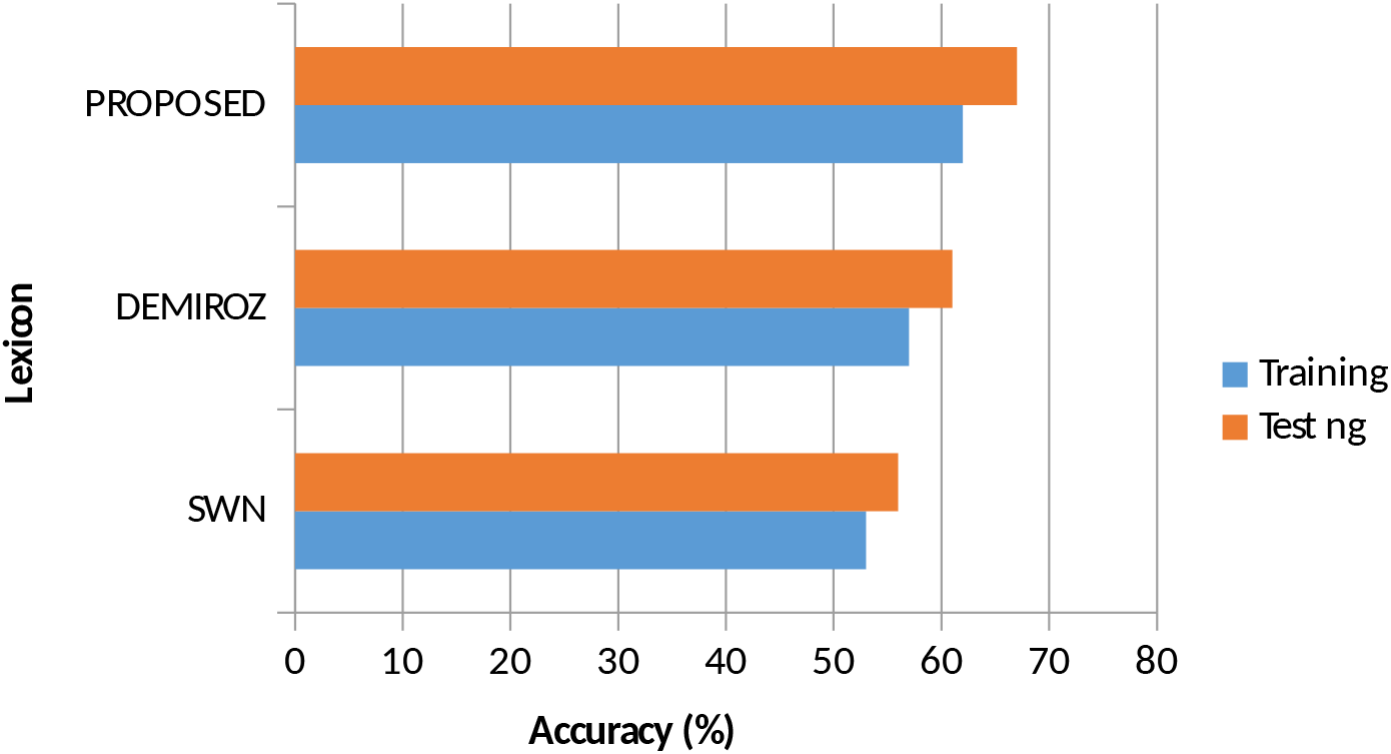
Accuracy comparison of Vote Flip Algorithm on each Lexicon.

The Fig 6 shows the accuracy results obtained by applying the vote flip algorithm on each of the comparing lexicon. We can observe that results are not very much high, with respect to general performances of sentiment analysis classifiers. This is because the algorithm we used is quite simple and it only keeps track of the sentiment terms. However, it shows the effectiveness of opinion terms for analyzing sentiments. Furthermore, our lexicon demonstrates promising results for both training and testing corpus.

Tables 5 and 6 show for each lexicon and each sentiment polarity, the precision, recall, and F-score on three datasets. We observe that our lexicon delivered better results for positive reviews than the negative or neutral regardless of the dataset. However, for neutral reviews there was a no improvement in the results, suggesting that the algorithm can further yield improved results, if proportion of neutral reviews was increased in the training dataset.

**Table 5:** Experimental results on training datasets (P = Precision, R = Recall, F = F-Score).

	Positive			Negative			Neutral		
Method	P	R	F	P	R	F	P	R	F
SWN	0.77	0.41	0.53	0.19	0.41	0.26	0.02	0.15	0.03
Demiroz	0.73	0.57	0.59	0.2	0.44	0.29	0.019	0.12	0.04
Proposed	**0.79**	**0.6**	**0.66**	**0.24**	0.44	**0.31**	0.011	0.04	0.003

**Table 6:** Experimental results on testing datasets (P = Precision, R = Recall, F = F-Score).

	Positive			Negative			Neutral		
Method	P	R	F	P	R	F	P	R	F
SWN	0.77	0.46	0.55	0.21	0.47	0.29	0.06	0.09	0.07
Demiroz	0.75	0.48	0.58	0.23	0.45	0.31	0.07	0.09	0.08
Proposed	**0.78**	**0.54**	**0.64**	**0.25**	0.46	**0.33**	**0.11**	0.07	0.006

On the testing dataset experiment, we can observe that proposed lexicon produces good results for both positive and negative reviews, which shows its effectiveness. The low neutral results depict that our algorithm does not consider the neutral reviews. A sentence is declared neutral if it contains equal distribution of positive and negative words. Moreover, the low neutral scores can be analyzed by the fact that we adopted a simple algorithm which does not take into account the semantic features in the sentences and considers only the opinion or non-opinion terms. However, enhanced algorithms for sentiment classification need to cope with semantic features in sentences.

### Lexicon Coverage

The algorithms produced the polarity lexicon that provides sufficient coverage to the domain-specific terms. The lexicon contains 1,314 terms, 65% positive, 30% negative, and 15% neutral. A majority of the terms (67%) pertaining to the drug and car dataset stored in the newly adapted lexicon are not present in the SWN. For example, ultrasound (+ve), frozen shoulder (-ve), heat spots (-ve), lazy eye (negative), side effect (-ve), were not covered. Among the words that belong to SWN, 71% have the different polarity class and score; a partial list of such words is presented in Table 3.

Majority of the differences are due to the connotation of terms in specific domain: *a repair* can be pleasant in general lexicon, but not for automobile matters; *growth* in drug domain mainly refers to ill enlargement of some organ due to chronic disease. The polarity differences between SWN and proposed domain dependent lexicon supports our assumption that domain dependent lexicon is appropriate for specific domains.

## Conclusions and Future Work

For the past several years, the OM applications are getting popular among online users for knowing about products, policies, and services. The OM has many applications including polarity classification, feature extraction, question answering, summary generation, and polarity lexicon construction.

Our aim in this study was to address the problem of constructing a domain-dependent polarity lexicon from a labeled set of reviews by adapting a domain-independent polarity lexicon to a specific domain. We studied and introduced polarity classification of a terms using domain-independent polarity lexicon, formulated an information theory model to derive domain-dependent polarity lexicon, presented a mathematical transformation into improved polarity modification method. We evaluated the performance of our proposed method on a simple sentence level polarity classification algorithm and measured the effectiveness of our approach by applying a number of metrics to compare the results with existing polarity lexicons and polarity modification methods. The results demonstrated that our proposed method can learn and assign the correct polarity scores to a new domain dependent polarity words.

The quantitative evaluation of the proposed method against the baseline methods shows that: (a) for a specific domain our method can provide a sufficient coverage of required opinionated text; (b) derived domain-specific lexicons have achieved improved performance in a real-world and manually built datasets; (c) polarity classification performance can be improved significantly with resulting adapted lexicon; and (d) threshold adjustment provides increased accuracy for the polarity classifications. The proposed framework is quite generalized and capable of classifying the opinionated text in any specific domain.

In the future, we will exploit semantic and contextual knowledge to classify the opinions in a better fashion. We also plan to incorporate context-aware features and evaluate the performance of other OM applications, such as multi-class opinion orientation and summarization. An automatic adjustment of threshold and weighting parameters in different domains with more lexicons will also be explored for optimal performance.

## References

[1] Turney, PD (2002) Thumbs Up or Thumbs Down?: Semantic Orientation Applied to Unsupervised Classification of Reviews, in the Proceedings of the 40th Annual Meeting on Association for Computational Linguistics, ACL ‘02, Morristown, US, 417–424.

[2] Gangemi A, Presutti V, Recupero D (2014) Frame-Based Detection of Opinion Holders and Topics: A Model and a Tool, Computational Intelligence Magazine, 9(1): 20–30.

[3] Xu X, Cheng X, Tan S, Liu Y, Shen H (2013) Aspect-level opinion mining of online customer reviews, Communications, 10(3): 25–41.

[4] Neviarouskaya A, Aono M (2013) Sentiment Word Relations with Affect, Judgment, and Appreciation, IEEE Transactions on Affective Computing, 4(4): 425–438.

[5] Moraes R, Valiati J, Gavio W (2012) Document-level Sentiment Classification: An Empirical Comparison between SVM and ANN, Expert Systems with Applications, 40(2): 621–633.

[6] Xia R, Zong C, Hu X, Cambria E (2013) Feature ensemble plus sample selection: A comprehensive approach to domain adaptation for sentiment classification, IEEE Intelligent Systems, 28(3): 10–18.

[7] Liu C, Hsaio W, Lee C, Lu G, Jou E (2012), Movie rating and review summarization in mobile environment, IEEE Transactions on Systems, Man, and Cybernetics, Part C: Applications and Reviews, 42(3):397–407.

[8] Demiroz G, Yanikoglu B, Tapucu D, Saygin (2012) Learning domain-specific polarity lexicons, Data Mining Workshops (ICDMW), IEEE 12^th^ International Conference on. IEEE, 674–679.

[9] Nielsen F (2011) A new ANEW: Evaluation of a word list for sentiment analysis in microblogs, arXiv preprint arXiv:1103.2903.

[10] Miller G (1995) Wordnet: A lexical database for English. Communications of the ACM, 38(11):39–41.

[11] Li X, Li J, Wu Y (2015) A Global Optimization Approach to Multi-Polarity Sentiment Analysis. PLoS ONE 10(4): e0124672 doi: 10.1371/journal.pone.0124672 25909740 10.1371/journal.pone.0124672 PMC4409395

[12] Baccianella, Stefano, Esuli A, Sebastiani F (2010) Sentiwordnet 3.0: An enhanced lexical resource for sentiment analysis and opinion mining, Proceedings of the 7th conference on International Language Resources and Evaluation (LREC’10) 10, 2200–2204.

[13] Choi Y, Cardie C (2009) Adapting a polarity lexicon using integer linear programming for domain specific sentiment classification, in Proceedings of the Conference on Empirical Methods in Natural Language Processing, 2(2):590–598.

[14] Lu Y, Castellanos M, Dayal U, Zhai C (2011) Automatic construction of a context-aware sentiment lexicon: an optimization approach, in Proceedings of the 20th international conference on World wide web, ser. WWW ‘11. New York, USA: ACM, 347–356.

[15] Martineau J, Finin T (2009) Delta TFIDF: An improved feature space for sentiment analysis, in Proceedings of the Third International ICWSM Conference, 258–261.

[16] Velikovitch L, Blair-Goldenshon S, McDonald R (2010) The viability of web-derived polarity lexicons, In Proceedings of Human Language Technologies of the North American Chapter of the Association for Computational Linguistics. Association for Computational Linguistics, 777–785.

[17] Lin Y, Zhang J, Wang X, Zhou A (2012) An information theoretic approach to sentiment polarity classification, in the proceedings of the 2nd Joint WICOW/AIRWeb Workshop on Web Quality ACM, 35–40.

[18] Fabbrizio G, Aker A, Gaizauskas R (2012) Summarizing Online Reviews Using Aspect Rating Distributions and Language Modeling, IEEE Intelligent Systems, 28(3): 28–37.

[19] Zhang D, Dong H, Yi J, Song L (2012) Opinion summarization of customer reviews, In proceedings of the International Conference on Automatic Control and Artificial Intelligence (ACAI 2012), 1476–1479.

[20] Das A, Bandyopadhyay S (2010) Topic-based Bengali Opinion Summarization, in Proceedings of 23rd Int’l Conf. on Computational Linguistics (COLING ‘10), 232–240.

[21] Ly D, Sugiyama K, Lin Z, Kan M (2011) Product Review Summarization From a Deeper Perspective, in Proceedings of 11^th^ Int’l Conf. Digital Libraries (ACM-IEEE/JCDL ‘ 11), 311–314.

[22] Rao D, Ravichandran D (2009) Semi-Supervised Polarity Lexicon Induction, In Proceedings of the 12th Conference of the European Chapter of the Association for Computational Linguistics, 675–682.

[23] Banea Carmen, Mihalcea R, Wiebe J (2008) A Bootstrapping Method for Building Subjectivity Lexicons for Languages with Scarce Resources, UNT Digital Library, 2764–2767.

[24] Asghar M, Khan A, Kundi F, Qasim M, Khan F, Ullah R, et al(2014). Medical Opinion Lexicon: An Incremental Model For Mining Health Reviews, International Journal of Academic Research, 6(1): 295–302.

[25] Denecke, Kerstin (2008) Using SentiWordNet for multilingual sentiment analysis, In proceedings of the IEEE 24th International Conference on Data Engineering (ICDEW), 507–512.

[26] Lu Y, Zhang P, Liu J, Li J, Deng S (2013) Health-related hot topic detection in online communities using text clustering. PLOS ONE 8(2): e56221 doi: 10.1371/journal.pone.0056221 . 23457530 10.1371/journal.pone.0056221 PMC3574139

[27] Chen, L., Wang, W., Nagarajan, M., Wang, S., Sheth, A. P. (2012). Extracting Diverse Sentiment Expressions with Target-Dependent Polarity from Twitter, In the Proceedings of the Sixth International AAAI Conference on Weblogs and Social Media(ICWSM), 50–57.

[28] Martineau, Justin, Finin T (2009) Delta TFIDF: An Improved Feature Space for Sentiment Analysis, in the Proceedings of the Third International ICWSM Conference, 258–261.

[29] Wilson Theresa, Wiebe J, and Hoffmann P (2009) Recognizing contextual polarity: An exploration of features for phrase-level sentiment analysis, Computational linguistics, 35(3): 399–433.

[30] Lorraine G, Na G, Kyaning W, Khoo C (2012) Sentiment lexicons for health-related opinion mining, in Proceedings of the 2nd ACM SIGHIT symposium on International health informatics, 219–226.

[31] Souza M, Vieira R, Busetti D, Chishman R, Alves I (2011) Construction of a Portuguese Opinion Lexicon from Multiple Resources, in the proceedings of the 8th Brazilian Symposium in Information and Human Language Technology (STIL’ 2011), 59–66.

[32] Mihalcea, R., C. Banea and J. Wiebe, 2007. “Learning Multilingual Subjective Language via Cross-Lingual Projections.” In Proceedings of the 45th Annual Meeting of the Association of Computational Linguistics, Prague, CZ, pp: 976–983.

[33] Bird S, Klein E, Loper E (2009) Natural language processing with Python, O’Reilly Media, Inc.

[34] Kundi F, Ahmad S, Khan A, Asghar (2014) Detection and Scoring of Internet Slangs for Sentiment Analysis Using SentiWordNet, Life Science Journal, 11(9):66–72.

[35] Taboada M, Brooke J, Tofiloski M, Voll K, Stede M (2001) Lexicon-based methods for sentiment analysis, Computational linguistics, 37(2): 267–307.

[36] Asghar M, Khan A, Ahmad S, Kundi F (2014) A Review of Feature Extraction in Sentiment Analysis, Journal of Basic and Applied Scientific Research, 4(3): 181–186.

[37] Ho S, Lieberman M, Wang P, Samet H. (2012) Mining future spatiotemporal events and their sentiment from online news articles for location-aware recommendation system, In the Proceedings of the First ACM SIGSPATIAL International Workshop on Mobile Geographic Information Systems, 25–32.

